# Can Patient Characteristics and Pre-Treatment MRI Features Predict Survival After Stereotactic Ablative Radiotherapy (SABR) Treatment in Hepatocellular Carcinoma (HCC): Preliminary Assessment

**DOI:** 10.3390/curroncol31100474

**Published:** 2024-10-19

**Authors:** Rachel Gravell, Russell Frood, Anna Littlejohns, Nathalie Casanova, Rebecca Goody, Christine Podesta, Raneem Albazaz, Andrew Scarsbrook

**Affiliations:** 1 Department of Radiology, Leeds Teaching Hospitals NHS Trust, Leeds LS9 7TF, UK; 2 Leeds Institute of Medical Research, Faculty of Medicine and Health, University of Leeds, Leeds LS2 9JT, UK; 3 Department of Oncology, Leeds Teaching Hospitals NHS Trust, Leeds LS9 7TF, UK

**Keywords:** hepatocellular carcinoma, radiotherapy, stereotactic techniques, machine learning, magnetic resonance imaging

## Abstract

Background: The study purpose was to develop a machine learning (ML)-based predictive model for event-free survival (EFS) in patients with hepatocellular carcinoma (HCC) undergoing stereotactic ablative radiotherapy (SABR). Methods: Patients receiving SABR for HCC at a single institution, between 2017 and 2020, were included in the study. They were split into training and test (85%:15%) cohorts. Events of interest were HCC recurrence or death. Three ML models were trained, the features were selected, and the hyperparameters were tuned. The performance was measured using Harrell’s C index with the best-performing model being tested on the unseen cohort. Results: Overall, 41 patients were included (training = 34, test = 7) and 64 lesions were analysed (training = 50, test = 14), resulting in 30 events (60% rate) in the training set (death = 6, recurrence = 24) and 8 events (57% rate) in the test set (death = 5, recurrence = 3). A Cox regression model, using age at treatment, albumin, and intra-lesional fat identified through MRI as variables, had the best performance with a mean training score of 0.78 (standard deviation (SD) 0.02), a mean validation of 0.78 (SD 0.18), and a test score of 0.94. Conclusions: Predicting the outcomes in patients with HCC, following SABR, using a novel model is feasible and warrants further evaluation.

## 1. Introduction

The inconspicuous nature of hepatocellular carcinoma (HCC) frequently results in late presentation and, consequentially, treatment is often palliative. For those with disease confined to the liver, orthotopic liver transplant or surgical resection remain the only curative treatment options for which there are stringent guidelines [1]. Patients who have advanced disease or do not meet the transplant criteria may benefit from non-surgical therapeutic options, such as trans-arterial chemo-embolisation (TACE), radiofrequency ablation (RFA), and/or systemic chemotherapy.

In recent years, additional treatment options have emerged, in particular stereotactic ablative body radiotherapy (SABR), a novel form of highly focused radiation treatment, which provides a high dose of radiotherapy concentrated on the tumour whilst limiting the dose to surrounding tissues, reducing toxicity. This technique was recently commissioned by the UK National Health Service (NHS) for use in the treatment of HCC [2]. It has previously been used successfully in patients with lung cancer (NHS England, 2020), prompting further exploration of its benefit in different cancer settings. The precise delivery afforded by SABR, delivering higher doses of radiotherapy per treatment fraction, whilst sparing adjacent tissue, also allows reduced treatment frequency [3]. Given that the predominant mode of death in those with HCC is liver failure, preserving function through localised liver-directed therapy is important. The delivery of therapeutic radiotherapy doses to HCC has historically been limited by the tolerance of the non-tumour liver cells to radiotherapy and the risk of radiation-induced liver disease. SABR enables the delivery of high radiotherapy doses to the tumour, with low rates of liver toxicity, in carefully selected patients, with preserved baseline liver function [4,5].

There have been developments in the potential utility of prediction models, which may help optimise treatment decision making based on patient characteristics, biochemical markers, and pre-treatment investigations. These models have demonstrated the ability to predict HCC recurrence post-transplant, based on patient-specific clinical parameters, with improved performance in models also encompassing magnetic resonance imaging (MRI) features [6]. Further studies focused on the incorporation of radiomic features report a potential benefit from improving the non-invasive diagnosis and prognostication of HCC [7].

The literature on SABR, specifically in regard to the treatment of HCC, continues to grow. Recent studies focused on predictive modelling have explored patient outcome measures and survival post-treatment, demonstrating an improvement in the local control of HCC post-SABR [8]. However, no papers, to date, have explored whether incorporating MRI imaging features is beneficial when predicting patient outcomes.

When focusing on different types of non-surgical treatments for HCC, predictive modelling, using both clinical features and radiomics, has been successfully investigated. For example, clinical radiomic models using MRI demonstrate good performance in patients undergoing their first TACE treatment [9] with further promising results in support of this using both MRI and CT [10]. Patient groups undergoing trans-arterial radioembolisation (TARE) also show encouraging results, where pre-treatment MRI radiomics and clinical features were used to predict the response to TARE [11]. Overall, incorporating a combination of both imaging radiomics and clinical features to predict the treatment response and outcome is more favourable than using just one subgroup of data [12].

Further published studies on SABR have focused on the survival benefit and its cost effectiveness in comparison to surgery and other established treatment methods. SABR is reported to perform at least as well as RFA [13]. Notwithstanding these promising initial results, along with the available evidence that is largely focused on the short-term benefits calls for further insight into long-term survival.

The aim of this study was to explore whether models based on patient characteristics, biochemical markers, and features derived from pre-treatment liver MRI could be used to predict event-free survival (EFS) post-SABR in patients with HCC. To the best of our knowledge, there are no published studies that have explored the utility of machine learning (ML) models to predict outcomes post-SABR for HCC, incorporating MRI imaging features specifically.

## 2. Materials and Methods

The transparent reporting of a multivariable prediction model for individual prognosis or diagnosis (TRIPOD) guidelines were adhered to during the study (Appendix A) [14].

### 2.1. Patient Selection

Formal ethics committee approval and informed written consent was waived for this study which was considered by the institutional review board to represent the evaluation of a routine clinical service.

**Figure 1:**
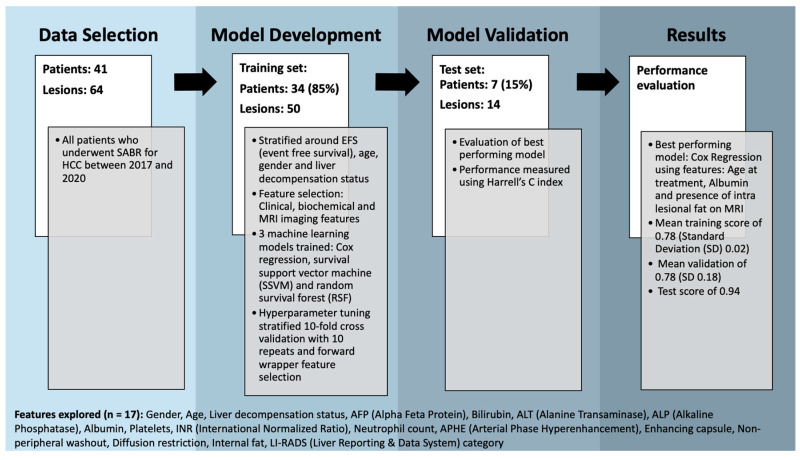
Flow diagram demonstrating methods with a breakdown of features explored.

Consecutive patients who underwent SABR treatment for HCC between 2017 and 2020 at a single large tertiary liver transplant centre in the UK (Leeds Teaching Hospitals NHS Trust) were retrospectively identified using institutional Electronic Patient Records (EPRs) and a radiotherapy departmental database. Data were collated on patient demographics, the presence (or absence) of underlying liver disease, biochemical markers and pre-treatment MR imaging features and manually recorded in a spreadsheet (Figure 1 shows a complete list of data collected).

Where patients presented with multiple liver lesions, the imaging features were stratified by each individual lesion rather than individual patients. Inclusion criteria were any patients > 18 years undergoing SABR for HCC. Patients who underwent prior or additional therapy for their lesions were included. Patients with incomplete datasets were excluded. Events of interest were HCC recurrence or death from any cause. If a patient had both, date of recurrence was used.

### 2.2. Image Acquisition and Analysis

MRI examinations were performed on one of three 1.5T scanners—2 Aera and 1 Avanto systems (Siemens^®^, Munich, Germany). The protocol included unenhanced sequences (Dixon with T1-weighted in- and out-of-phase sequence, half Fourier acquisition single-shot turbo spin echo (HASTE) T2-weighted sequence, T2 turbo spin echo (TSE) balanced steady-state free precession line acquisition with undersampling (BLADE) fat saturation), diffusion weighted imaging (DWI) [b values 50, 200, 500, 750] and volumetric interpolated breath-hold examination (VIBE) T1-weighted fat suppressed sequence and contrast enhanced dynamic and hepatobiliary phase imaging using the VIBE sequence. Post-contrast VIBE T1-weighted fat suppressed sequences were obtained following bolus-tracked intravenous injection of 10 mL of gadoxetic acid (Gd-EOB-DTPA, Primovist^®^, Bayer HealthCare, Hong Kong, China) by a power injector at a rate of 1.5 mL/s followed by a 20 mL saline flush. Consequently, timings varied but were at approximately 25 s, 60 s, 2 min, 10 min and 20 min post contrast.

Using MRI characteristics, each tumour was classified using the liver imaging and reporting data system (LI-RADS). LI-RADS is a standardised liver lesion imaging classification method used to predict the risk of HCC [15] in those who are at high risk, over 18 years of age with liver cirrhosis. This method produces a risk score from 1 to 5, and lesions scoring LI-RADS 4 or above raise concern for HCC. In addition to the LI-RADS score, diffusion restriction and internal fat content were also assessed on imaging. Please see Table 1 for more detailed breakdown of the LI-RADS scoring system and an explanation of the features. These lesion imaging characteristics were initially evaluated by a team of specialist hepatobiliary radiology consultants and discussed in a multidisciplinary team if needed. For this study, each scan was re-evaluated and classified by a board-certified hepatobiliary radiologist with >10 years’ experience in oncological liver MRI.

### 2.3. Machine Learning Framework and Statistical Analysis

Python (version 3.9.12) was used to implement the machine learning framework. Patients were split into a training cohort and an unseen test cohort (85%:15%) stratified around age, gender, hepatic decompensation, the size of the largest lesion and EFS. The split was carried out at the patient level rather than at the lesion level to avoid data leakage. Significant differences between the demographics of the training and test groups were assessed using a *t*-test for continuous data and a χ^2^ test for categorical data (scipy v1.9.3). A *p*-value of <0.05 was regarded as significant.

Overall, 17 different baseline clinical, biochemical and MRI-based imaging features were investigated with correlated features removed (Spearman correlation > 0.8). These features are outlined in Figure 1. Categorical data were dummy encoded (pandas v1.5.2) and a standard scaler was fitted and transformed to the training set before being applied to the test set (scikit-learn v0.24.2).

Three ML models (cox regression, survival support vector machine (SSVM) and random survival forest (RSF)) were trained (scikit-survival v0.20.0), features were selected, and hyperparameters were tuned using a stratified grid search with 10-fold cross-validation with 50 repeats and forward wrapper feature selection (scikit-learn v0.24.2). A maximum of three features were chosen for each model; this was due to rough rule of thumb of having one feature per 10 events within the training cohort. The cross-validation was stratified around one event. Where a cross-validation split resulted in a patient having lesions in both the training and validation datasets, the lesions relating to this patient were all moved to the validation dataset. This was performed using a custom cross-validation class to minimise data leakage, where clinical features were being assessed. As a comparator, a simple cox regression model based on the LI-RADS score was also created using the same cross-validation methodology. As the dataset consisted of only LI-RADS 4 and 5 lesions, this was treated as a binary feature; i.e., whether the lesion was LI-RADS 5 or not.

Model performance was assessed using the Harrell’s C index with the best performing model being tested once on unseen test data. Kaplan–Meier survival curve analysis was evaluated for the best-performing model on the test data.

## 3. Results

Overall, 41 patients were included after both inclusion and exclusion criteria were applied. A total of 45 patients underwent SABR between 2017 and 2020. Complete datasets were unavailable for four patients, and as a result, these patients were excluded from the study. The study cohort mean age was 70 years (range 30–89 years) with a male majority of 32 patients (78%) to 9 female patients. Twenty (49%) patients had more than one hepatic lesion present, but the median number of lesions was one (range 1–3) with 10 patients presenting with macrovascular invasion. The most common aetiologies of underlying liver disease were alcohol excess (n = 20), viral disease inclusive of hepatitis B and C (n = 11) and non-alcoholic fatty liver disease (NAFLD) (n = 7). Some patients had concurrent underlying pathologies such as alcohol-related liver cirrhosis and viral hepatitis. Patient demographics are outlined in Table 2.

The patients were split into training (n = 34) and test sets (n = 7) of which 64 individual lesions were analysed (training = 50, test = 14). This resulted in 30 events (60%) in the training set (death = 6, recurrence = 24) and 8 events (57%) in the test set (death = 5, recurrence = 3). The median follow-up time was 11 months (range 1–33 months) for the training set and 11 months (1–19) for the test set.

The basic cox regression model created using the LI-RADS score had a mean training C index of 0.61 (standard deviation (SD) 0.02) and a mean validation C index of 0.61 (SD 0.14). A cox regression model derived from age at treatment, presence of fat within the lesion (on pre-treatment MRI) and albumin level was the best performing with a mean training C index of 0.78 (SD 0.02), a mean validation of 0.78 (SD 0.18) and a test score of 0.94. The SSVM model was the next best performing model, which was derived using age at treatment, neutrophil concentration and alkaline phosphatase (ALP) with a mean training score of 0.78 and mean validation score of 0.74. The third model, RSF, used age at treatment and international normalised ratio (INR) level producing a mean training score of 0.85 and mean validation score of 0.75. Please see breakdown of results in Table 3.

**Figure 2:**
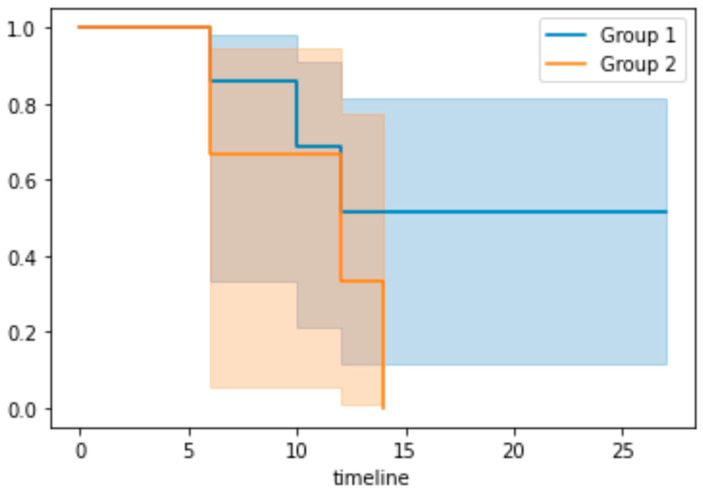
Kaplan–Meier survival curve. *X* axis: timeline in months. Group 1 represents the low-risk group and group 2 represents the high-risk group based on the median predictive risk score from the training dataset.

A Kaplan–Meier survival curve (Figure 2) was generated for the cox regression model on the test data. Patients were grouped according to their risk features based on the median predictive score from the training data. Group 1 contained patients with a low-risk score, whereas group 2 represented those with a high-risk score. It showed a superior survival rate for those in the low-risk group. Due to the small dataset available, the 95% confidence intervals were wide.

## 4. Discussion

Overall, 41 patients (64 lesions) with HCC treated with SABR underwent analysis, and 17 features were explored encompassing pre-treatment clinical, biochemical and MRI parameters. Of these parameters, patient age, albumin level and the presence of fat within the lesion (determined on MRI) demonstrated the best performance for EFS prediction using a cox regression model. This model gave a mean training AUC of 0.78 (SD 0.02), a mean validation of 0.78 (SD 0.18) and a test score of 0.94. Kaplan–Meier survival statistics were carried out on the best-performing model for the test data, which demonstrates a superior survival rate for those in the low-risk group. It must be acknowledged that due to the small sample size, we could only report with wide 95% confidence intervals. In summary, this indicates that there is the potential to predict outcomes of patients with HCC following treatment with SABR using cox regression predictive modelling that incorporates both clinical and MRI imaging features. To the best of our knowledge, this is the first study evaluating predictive modelling in patients undergoing SABR treatment for HCC specifically using MRI features and clinical parameters.

SABR is an established and well-known treatment for lung cancer, in particular small cell lung cancer, and the literature highlights success in radiomics and predictive modelling for SABR in this cohort [16,17]. The use of radiomics and deep learning algorithm techniques have also been introduced in the field of hepatology to predict outcomes in those undergoing transjugular intrahepatic portosystemic shunts (TIPS) with promising results [18,19]. Further, radiomics has also been used in predictive modelling with success in patients undergoing non-surgical treatment for HCC [9,10,11]. Given these findings, the predictive value of radiomics in HCC patients undergoing SABR should be a focus of future work.

In relation to HCC, previous studies have focused on predictive modelling for longstanding and established treatments for HCC that are both surgical and systemic. Predictive modelling has been used to successfully predict HCC recurrence using pre-treatment MRI features [6], and a large dual centre study [20] described models predicting mortality, non-local recurrence and also adverse outcomes secondary to radiotherapy treatment. Although the study by Chamseddine et al. [20] reports on radiotherapy treatment, our study design and findings share several common features. Both of our cohorts were an unselected, continuous group of patients with largely comparable demographics, and the ML models used similar data points to formulate the algorithms. Our study, however, was the first of this kind that we are aware of to use imaging features as part of the machine learning model. The study by Chamseddine [20] had a significantly larger dataset (207 test cohort and 143 validation cohort), yet our modelling showed similar outcomes (cox regression model EFS AUC 0.78 vs. 0.74). Generating comparable findings in a similar, larger cohort further supports the potential applicability of ML models in predicting outcomes and determining risk in this cohort.

The literature to date along with the results of this preliminary study shows promise for the future prediction of outcomes or event-free survival in SABR patients. However, a recently published large systematic review of prognostic models for outcome prediction highlights a need for a greater degree of external validation [21] in this clinical scenario.

Driving changes in practice can help better target resources and improve the patient journey. Imaging features of HCC are important in determining event-free survival along with patient and biochemical factors with further research needed focusing on imaging features alone given these results.

## 5. Limitations

Limitations to this study include a small sample size as well as the single-centre retrospective nature of the study. SABR is an emerging treatment for HCC, and consequently cohort sizes are restricted, but as more patients receive this therapy, larger studies can be conducted. This is presumed secondary to its relatively new introduction in the UK as a therapy for this disease. Further to this, a greater number of large studies will allow centres to externally validate the initial findings on larger multi-centre dataset.

The analysis of individual patients rather than lesions may be favourable in future studies to help reduce confounding factors. In this preliminary study, data collection and ML methods meant that individual lesions were best analysed; however, a greater number of lesions do affect outcome, and thus focusing on individual patients in the future is beneficial for accuracy. A combination of small sample size with a focus on lesions also affects the Kaplan–Meier reliability. Further, there was a lack of external validation of this model to assess generalisability; this was beyond the scope of this initial study but will be a focus of future planned work.

In addition, our model, whilst incorporating both clinical and imaging features has not evaluated radiomic features in part due to the risk of false discovery in a small dataset when using many parameters. The incorporation of radiomic features into a prediction model will be a subject of future study.

## 6. Conclusions

ML models have successfully been used to previously predict outcomes in patients with cancer. This preliminary study evaluated the use of prediction models on patients undergoing SABR for HCC, which is a relatively novel form of treatment aimed at patients with incurable malignancy. To our knowledge, our paper is the first to incorporate MRI imaging features specifically in this patient population to predict outcome.

In conclusion, outcome prediction for patients with HCC undergoing SABR using a model encompassing clinical and imaging-specific features is feasible. Further validation in a larger multi-centre cohort will be imperative to confirm initial findings before clinical translation.

## Figures and Tables

**Table 1:** Description of MRI imaging features evaluated.

MRI Imaging Features	Description	LI-RADS or Ancillary Features
Arterial phase hyperenhancement (APHE)	Lesion enhancement on arterial phase imaging is greater than enhancement of the background liver	LI-RADS major feature
Non-peripheral washout	On portal venous or delayed phased imaging, the mass is of decreased attenuation when compared to that of the background liver	LI-RADS major feature
Capsule enhancement	A peripheral, smooth rim of hyper enhancement surrounding the lesion on portal venous or delayed phased imaging	LI-RADS major feature
Diffusion restriction	The lesion demonstrated diffusion restriction on DWI and ADC sequences	Additional imaging feature
Internal tumour fat	Foci of fat within the mass	Additional imaging feature

**Table 2:** Outline of patient demographics.

Patient Demographics	
Total included participants (n)	41
Gender (male, female)	32, 9
Mean age (mean, range)	71 (30–89)
Max lesion diameter (mean, range, cm)	2.7 (0.8–5.8)
No of lesions (n, range)	1.5 (1–3)
1 (n)	21
2 (n)	17
3 (n)	3
Vascular invasion (n, %)	10 (24)

**Table 3:** Hyperparameters, selected features, training and validation scores for the best performing machine learning models, (survival support vector machine (SSVM), and random survival forest (RSF)).

Model	Hyperparameters	Features	Mean Training Score	Mean Validation Score	Harrell’s C Index
Cox Regression	{‘alpha’: 13.876647909813085}	[‘Age at treatment’, ‘Internal fat_N’, ‘Albumin (g/L)’]	0.78 (0.02)	0.78 (0.18)	0.78
SSVM	{‘alpha’: 0.001, ‘optimizer’: ‘avltree’}	[‘Age at treatment’, ‘neutrophils’, ‘ALP (iU/L)’]	0.78 (0.02)	0.74 (0.19)	0.76
RSF	{‘bootstrap’: True, ‘max_depth’: 2, ‘max_features’: ‘sqrt’, ‘min_samples_leaf’: 5, ‘min_samples_split’: 5, ‘n_estimators’: 5}	[‘Age at treatment’, ‘INR’]	0.85 (0.02)	0.75 (0.21)	0.80

## Data Availability

The dataset presented in this article is not readily available because due to data protection laws.

## Appendix A

**Table A1:** TRIPOD checklist.

Section/Topic	Item	Checklist Item	Page
**Title and abstract**			
Title	1	Identify the study as developing and/or validating a multivariable prediction model, the target population, and the outcome to be predicted.	1
Abstract	2	Provide a summary of objectives, study design, setting, participants, sample size, predictors, outcome, statistical analysis, results, and conclusions.	1 *
**Introduction**			
Background and objectives	3a	Explain the medical context (including whether diagnostic or prognostic) and rationale for developing or validating the multivariable prediction model, including references to existing models.	1,2 *
	3b	Specify the objectives, including whether the study describes the development or validation of the model or both.	1,2 *
**Methods**			
Source of data	4a	Describe the study design or source of data (e.g., randomised trial, cohort, or registry data), separately for the development and validation datasets, if applicable.	3,4 *
	4b	Specify the key study dates, including start of accrual; end of accrual; and, if applicable, end of follow-up.	3,4 *
Participants	5a	Specify key elements of the study setting (e.g., primary care, secondary care, general population) including number and location of centres.	3,4 *
	5b	Describe eligibility criteria for participants.	3,4 *
	5c	Give details of treatments received, if relevant.	3,4 *
Outcome	6a	Clearly define the outcome that is predicted by the prediction model, including how and when assessed.	3,4,5 *
	6b	Report any actions to blind assessment of the outcome to be predicted.	NA
Predictors	7a	Clearly define all predictors used in developing or validating the multivariable prediction model, including how and when they were measured.	3,4,5 *
	7b	Report any actions to blind assessment of predictors for the outcome and other predictors.	NA
Sample size	8	Explain how the study size was arrived at.	3 *
Missing data	9	Describe how missing data were handled (e.g., complete-case analysis, single imputation, multiple imputation) with details of any imputation method.	3,4,5
Statistical analysis methods	10a	Describe how predictors were handled in the analyses.	NA
	10b	Specify type of model, all model-building procedures (including any predictor selection), and method for internal validation.	4,5 *
	10d	Specify all measures used to assess model performance and, if relevant, to compare multiple models.	4,5
Risk groups	11	Provide details on how risk groups were created, if applicable.	NA
**Results**			
Participants	13a	Describe the flow of participants through the study, including the number of participants with and without the outcome and, if applicable, a summary of the follow-up time. A diagram may be helpful.	NA
	13b	Describe the characteristics of the participants (basic demographics, clinical features, available predictors), including the number of participants with missing data for predictors and outcome.	5,6 *
Model development	14a	Specify the number of participants and outcome events in each analysis.	5,6 *
	14b	If completed, report the unadjusted association between each candidate predictor and outcome.	NA
Model specification	15a	Present the full prediction model to allow predictions for individuals (i.e., all regression coefficients, and model intercept or baseline survival at a given time point).	NA
	15b	Explain how to the use the prediction model.	NA
Model performance	16	Report performance measures (with CIs) for the prediction model.	5,6 *
**Discussion**			
Limitations	18	Discuss any limitations of the study (such as nonrepresentative sample, few events per predictor, missing data).	7,8 *
Interpretation	19b	Give an overall interpretation of the results, considering objectives, limitations, and results from similar studies, and other relevant evidence.	6,7 *
Implications	20	Discuss the potential clinical use of the model and implications for future research.	6,7 *
**Other information**			
Supplementary information	21	Provide information about the availability of supplementary resources, such as study protocol, Web calculator, and datasets.	NA
Funding	22	Give the source of funding and the role of the funders for the present study.	8

Comments as denoted by * in the tripod checklist.

- See title (**Title**).
- This study is a preliminary investigation into whether patient characteristics and pre-treatment investigations can predict survival after stereotactic ablative radiotherapy (SABR) treatment in hepatocellular carcinoma (HCC).
- See abstract for all required information (**Abstract**).
- The introduction describes the current issues with treatment of HCC and the poor outcomes for these patients. We introduce the possibility that a ML model can help better predict which patients will benefit from the more novel SABR management. ML models have been shown they can predict outcomes in patients with HCC who undergo a transplant but no models exist for treatment with SABR. This study describes both development and validation cohorts (**Introduction**).
- The data from the study were obtained from consecutive patients undergoing SABR treatment at a single tertiary centre between 2017 and 2020. The median follow-up period was 11 months (1–33 months) (**Patient selection**).
- Inclusion criteria were any patients >18 years, undergoing SABR for HCC. Patients who underwent prior or additional therapy for their lesions were included. Exclusion criteria were the presence of an incomplete dataset.
- Events of interest were HCC recurrence or death from any cause. If a patient had both, date of recurrence was used (**Patient selection**).
- Predictors used to develop the ML tool are outlined in Figure 1 (**Methods**).
- Sample size included all patients who underwent SABR during the specified time period (**Methods**).
- Three ML models (cox regression, survival support vector machine (SSVM) and random survival forest (RSF)) were trained (scikit-survival v0.20.0), features were selected and hyperparameters were tuned using a stratified grid search with 10-fold cross-validation with 50 repeats and forward wrapper feature selection (scikit-learn v0.24.2). A simple cox regression model based on the LI-RADS score was also created using the same cross-validation methodology to be used as a comparator (**Methods**).
- Model performance was assessed using Harrell’s C index with the best-performing model being tested once on unseen test data. Kaplan–Meier survival curve analysis was evaluated for the best-performing model on the test data (**Methods**).
- Overall, 45 patients underwent SABR during the study period, but four were excluded due to incomplete data. The mean age was 70 years with 32 male (78%) and 9 female patients, of whom 34 patients were included in the training set and 7 in the test set. There were 30 events (60%) in the training set (death = 6, recurrence = 24) and 8 events (57%) in the test set (death = 5, recurrence = 3) (**Results**).
- The basic cox regression model created using the LI-RADS score had a mean training C index of 0.61 (standard deviation (SD) 0.02) and a mean validation C index of 0.61 (SD 0.14). The ML cox regression model was the best performing with a mean training C index of 0.78 (SD 0.02), a mean validation of 0.78 (SD 0.18) and a test score of 0.94. (**Results**).
- Limitations to this study include a small sample size as well as the single-centre retrospective nature of the study. Another limitation is the lack of external validation of this model to assess generalisability; this was beyond the scope of this initial study but will be a focus of future planned work (**Discussion**).
- The results of this preliminary study shows there is scope for the use of ML models to help with the prediction of outcome or event-free survival in patients with HCC undergoing SABR treatment (**Discussion**).
